# Secondary Metabolites Isolated from the Genus *Psammocinia* Sponges: Mapping Their Chemistry and Biological Activities

**DOI:** 10.3390/md24040132

**Published:** 2026-04-01

**Authors:** Dele Abdissa Keneni, Tarryn Swart, Alyson Bennett, Michelle Isaacs, Rosemary Dorrington

**Affiliations:** 1 Department of Chemistry, Rhodes University, Makhanda 6140, South Africa; 2 Department of Chemistry, College of Natural Sciences, Jimma University, Jimma P.O. Box 378, Ethiopia; 3 Department of Biochemistry, Microbiology and Bioinformatics, Rhodes University, Makhanda 6140, South Africa; tarrynswart10@gmail.com (T.S.); alyson.bennett@ru.ac.za (A.B.); m.isaacs@ru.ac.za (M.I.); r.dorrington@ru.ac.za (R.D.); 4 South African Institute for Aquatic Biodiversity, Makhanda 6140, South Africa

**Keywords:** *Psammocinia*, *Ircinianins*, terpenoids, indole alkaloids, and secondary metabolites

## Abstract

This review paper covers publications from 2013 to July 2025, and describes brominated and non-brominated indole alkaloids, ircinianins, terpenoids, and polyketide compound classes from the marine sponge of the genus *Psammocinia*. It provides an overview of the reported secondary metabolites, their source organisms, geographic origins, and associated biological activities. Also, the structure-activity relationship study and biosynthetic pathways of the reported compounds are illustrated. Herein, 15 new secondary metabolites, including 11 terpenoids and four akaloids, were identified in the *Psammocinia* sponge species during this period. Briefly, the biological activities of these secondary metabolites involve molecular, cellular, and microbial targets.

## 1. Introduction

It is estimated that oceans cover over 70% of the Earth’s surface and represent about 95% of the biosphere ecosystem that harbours many world macro and microbiota [1,2,3]. The marine environment embraces a rich source of unique bioactive compounds with rich chemical diversity [4]. Marine sponges (Phylum Porifera) produce numerous bioactive secondary metabolites, also known as natural products (NPs), with exceptional carbon skeletons that differ from those of terrestrial organisms. Due to their notable structural diversity and wide range of biological activities, these metabolites have become a promising source of new therapeutic agents for treating diseases [5,6]. Due to high chemical diversity refined through evolutionary selection, NPs are suitable for interacting efficiently with biomolecular binding sites [7], and offer boundless opportunities for drug discovery [8,9] as the primary source of drugs used in modern medical treatment [10,11,12]. NPs originate as secondary metabolites from various origins, most notably plants, marine microfauna, and microorganisms [13]. They have shown potential to combat various diseases, including cardiovascular, cancer, and infectious diseases [12]. Mehbub et al., (2016) [14] reviewed marine natural products (MNPs) derived from sponges of the order *Dictyoceratida* between 2001 and 2012, highlighting their potential as promising candidates for drug discovery, and contributing to more than 20% of all newly discovered sponge-derived compounds, making it the most prolific among all sponges. Next, Abdelaleem et al., (2020) [15] extended this work by reviewing MNPs from the order Dictyoceratida, covering the period from 2013 to 2019.

In this review, the databases of Google Scholar, ScienceDirect, Springer, Scopus, and PubMed were utilized in the search for NPs isolated from *Psammocinia* sp. and their associated biological activities. The biological activity is summarized to showcase the biological relevance of these compounds in drug discovery and development. It is designed to compile key elements for understanding the chemistry and bioactivity of NPs derived from *Psammocinia* sp. We presented each compound by its class, chemical structure, place of sample collection, and the reported bioactivities (Supplementary Information, SI Table 2). However, morphological identification of *Psammocinia* species presents certain challenges. Robinson et al., (2007) [16] noted that distinguishing *Psammocinia* from closely related genera, such as *Ircinia* and *Cacospongia*, can be challenging.

### 1.1. Challenges in the Morphological Identification of Psammocinia sp.

Species of the genus *Psammocinia* are difficult to differentiate from closely related species of the genus *Ircinia*, as both genera contain debris in fibres. *Psammocinia* species are distinguished by their sand-armoured surface and skeletal morphology, which features a regular, reticulate fibre skeleton [17]. The name *Psammocinia* was initially proposed as a subgenus of *Ircinia* by Lendenfeld in 1889 and later recognized as a genus by Bergquist in 1980. However, distinguishing between certain *Ircinia* and *Psammocinia* species remains problematic, as both share core primary fibres embedded with sand particles [18]. Sim et al., (2017) [18] conducted a morphological study to identify feature distinctions between *Psammocinia* and *Ircinia* collected from Jeju Island, Ulleung Island, and Gageo Island in the Republic of South Korea between 1998 and 2015, at depths of 15–30 m using SCUBA diving, primarily building on the previous work of Cook and Bergquist (1998) [17]. Sim and his co-worker found that in *Psammocinia* specimens, the sand grains within the primary fibres are generally larger than those in *Ircinia*. In addition, the secondary fibres of *Psammocinia* are mostly cored with sand, while those of *Ircinia* typically lack sand. The surface conules of *Ircinia* are usually low and unclear, whereas those of *Psammocinia* are visible and distinct [18].

In previous work by Sim et al., (2017) [18] *P. chupoensis* was incorrectly recorded under the genus *Ircinia* based on skeletal structure. However, it was noted that *P. chupoensis* has both primary and secondary fibres cored with sand throughout the sponge, unlike *Ircinia*, whose secondary fibres lack sand. Based on these appearances, Kim et al., (2018) [19] reclassified *Ircinia chupoensis* as *P. chupoensis*. The genus *Psammocinia* is characterized by thin, threadlike fibres of clear, unpigmented spongin that occur freely in bundles or masses outside the primary fibre skeleton. While this feature is like *Ircinia*, *Psammocinia* is distinguished by a sand coat, a continuous surface layer composed of tightly packed sand grains, and therefore exhibits distinct morphological features from those of *Ircinia* [19]. 

Recently, another reclassification was made to a *Psammocinia alba* from Sodwana Bay, South Africa, collected at 25 m as a new species rather than a junior species of the Indonesian *P. alba* [20]. The distinguishing feature was the thickness of the primary and secondary fibres, 150–210 µm vs. 80 µm and 51–98 µm vs. 20 µm, respectively, for *P. samaai* compared to *P. alba* [20]. 

Morphological plasticity and cryptic diversity increase misidentification risks in marine sponges. Integrative taxonomy, DNA barcoding, improves species delimitation. Accurate identification is crucial in chemotaxonomy because taxonomic errors can mislead compound attribution and disrupt biogeographic and evolutionary interpretations [21]. Thus, robust integrative taxonomy is essential for accurate NP mapping and reproducibility in NP drug discovery research.

To morphologically identify sponge specimens, the effective use of sponge spicules in taxonomy, ecology, and environmental research requires detailed knowledge of their shapes and their patterns of distribution among sponge species [22]. Yet, from the perspective of this review, the morphological identification of *Psammocinia* sp. remains inadequate, likely because various sponges of the order *Dictyoceratida* lack spicule morphology and skeletal structures, making it difficult to use the traditional morphological tools to distinguish them. The class Demospongiae, which accounts for 83% of all recognized living sponges, is the largest and most morphologically diverse within the phylum Porifera. Their skeletons are typically composed of opaline silica spicules, often reinforced with spongin fibres, although in some species the skeleton consists only of spongin or chitin fibres [23]. However, despite the high variation, assigning specific spicule morphotypes to specific taxa remains challenging, as the relationship between spicule types and sponge taxa is rarely straightforward [22]. Most importantly, recent studies emphasize integrative approaches to species identification, using a combination of morphology, DNA sequencing, and ecological context to achieve more precise species delimitation. Particularly, the most widely used alternative method for species identification is DNA barcoding, which distinguishes species based on specific nucleotide sequences [24]. 

Therefore, the challenges in the morphological identification of *Psammocinia* sp. could be addressed through the modern era of morphological studies. As reviewed by Ford et al., (2023) [25] the recent morphological analyses rely on several key techniques: descriptive or qualitative data from gross dissections and histology; quantitative data from linear and geometric morphometrics; and digital scanning, which may be supplemented with traditional morphological analysis [25] and molecular analysis/DNA sequencing [24]. These methods are mainly used for descriptive work, including two-dimensional (2D) illustrations (e.g., line drawings and photographs) and three-dimensional (3D) features (photogrammetry, which uses digital images) [25].

### 1.2. Taxonomic and Geographic Distribution of Psammocinia sp.: Overview 

Taxonomic novelty has a high potential for providing novel NPs discoveries [26], and species belonging to specific taxonomic groups and geographical regions may serve as valuable sources of NP with potential bioactivity [27]. The order *Dictyoceratida* comprises six families, 41 genera, and a total of 487 species [28]. The genus *Psammocinia* (family *Irciniidae*, order *Dictyoceratida*) was originally introduced as a subgenus of *Hircinia* and later reclassified under *Ircinia* by Lendenfeld in 1889, with species sharing morphological features typical of *Ircinia* [29]. According to Lendenfeld, the genus *Psammocinia* was later expanded to include 23 species, distinguished by a dermis reinforced with a thick crustose layer of foreign material [21]. However, biochemical analysis shows that the sesterterpene tetronic acid variabilin chemotype has been identified exclusively in the genera *Ircinia*, *Psammocinia*, and *Sarcotragus* [29]. 

**Figure 1:**
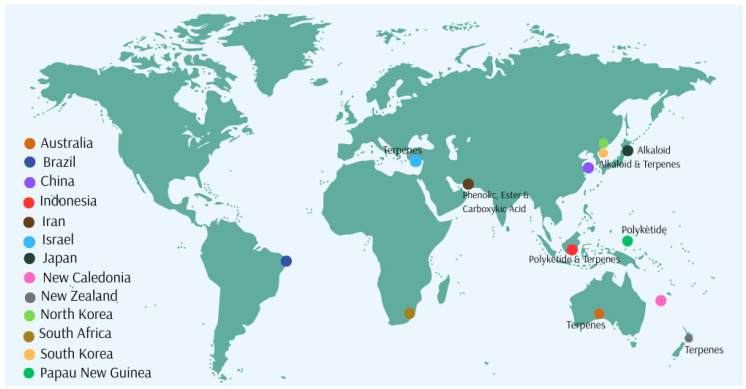
Global distribution of *Psammocinia* species and the chemical diversity of compound classes, with countries highlighted in different colours. Note: Species from Brazil, China, North Korea, New Caledonia, and South Africa lack studies involving the isolation, characterization, or bioactivity evaluation of secondary metabolites in this review period.

Taxonomic records indicate that, earlier to 1995, only five species of *Psammocinia* had been identified, including *P. aff. bulbosa* [16,29]. Of these, *P. beresfordi* n. sp., *P. hawere* n. sp., and *P. verrucosa* n. sp. were described from the Northland region of New Zealand [29]. Cook and Bergquist (1998) [17] revised the genus *Psammocinia*, noting that the waters around New Zealand and the southeastern coast of Australia have served as centres of diversification for the genus. In addition, they reported the existence of several undescribed species, including a few from South Africa and at least one from Japan. According to Schönberg et al., (2017) [30] a total of 25 *Psammocinia* species have been identified. The majority have been described from New Zealand and South Korea, with only a single species reported from Brazil (Figure 1). However, as of 2018, a total of 33 species of *Psammocinia* had been reported worldwide, of which 17 species are described from Korean waters [18,19,31]. 

Yet, according to the online World Porifera Database (WPD), approximately 38 species of *Psammocinia* (SI Table 1) have been recognized [32]. Among these, *P. alba* obtained at a depth of 25 m, and *P. cf. arenosa* at a depth of 12–34 m were identified by Samaai et al., (2019) [33] from a specimen collected in Sodwana Bay, north-eastern coast of South Africa [20,31]. The global distribution, chemical diversity, and number of biologically active compounds isolated from the genus *Psammocinia* (SI Table 2), which are relevant to various areas of drug discovery, served as the primary motivation for conducting this review.

Chemical biogeography seeks to explore how the spatial distribution of organisms is reflected in their chemical diversity, providing unique insights into the ecological, evolutionary, and environmental factors that shape and sustain NP profiles across regions [34]. Variation in the geographic distribution of marine sponges can lead to differences in the biological activity of their secondary metabolites [35]. 

Metabolic variation observed in samples from different locations may be reflected in their bioactivity [36]. As the review by Bayona et al., (2020) [35] indicated, secondary metabolites isolated from marine organisms often contain distinctive structural features, and many of them have demonstrated diverse bioactivities. Despite a large geographical separation, sponges can share a common metabolic framework in terms of qualitative features [35,36] but may exhibit different biological activities. This variation is likely influenced by environmental factors (depth, nutrients, pH, temperature, and pressure). According to Bayona et al., (2020) [35], geographical location was identified as one of the most influential factors contributing to variation in secondary metabolites in sponges. 

### 1.3. Chemical Diversity 

This paper follows earlier reviews by Mehbub et al., (2016) [14], covering the period 2001–2012, where a total of 559 new compounds were reported, followed by Abdelaleem et al., (2020) [15], covering 2013–2019, where a total of 195 new compounds and 189 known compounds were isolated from various genera within the order *Dictyoceratida*. Notably, 149 compounds were reported in 2018 and 136 in 2017, targeting different biological activities [15]. Therefore, Abdelaleem et al., (2020) [15] review revealed that terpenes were the predominant (73%) chemical class, which includes simple terpenes, diterpenes, sesquiterpenes, sesterpenes, and other terpenoid scaffolds. Importantly, 54% of the compounds described during this period were newly isolated, while 44% were previously known. Nitrogenous compounds, including alkaloids and alkaloid-related structures, comprised 13% of the reported classes [15]. *Psammocinia* sp. have been identified as the source of furanosesterterpenes, dimethyl sulphide, methyl isocyanide, methyl isothiocyanate, steroids, sphingolipids, hydroquinone, cyclic hexapeptide, and psymberin (*Irciniastatin* A) [14]. In addition, furanosesterterpene tetronic acids, sulawesins A-C, polybrominated phenyl ethers, chlorinated amino acid derivatives, and polyhydroxy steroids have previously been investigated for their various biological activities [15]. A summary of the chemically investigated *Psammocinia* sponges, along with their chemical classes, biological activities, and geographical locations, has been reviewed (SI Table 2).

#### 1.3.1. Indole Alkaloids 

*Psammocinia* sponges are recognized as a source of indole alkaloids [6], which are characterized by their unique structural features and diverse biological activities, offering a rich source of novel chemical entities [37]. For instance, a specimen of *Psammocinia* collected at a depth of 5 m via SCUBA diving in Amakusa, Japan, yielded a new methylenedioxy dibromoindole, amakusamine (**1**) [37]. 

Compound **1** was derivatized into 16 analogues, exhibiting varying growth inhibition against RANKL at 50 µM. This implies the importance of the structure-activity relationship (SAR) study in the pursuit of biological activity. Essentially, SAR signifies the correlation between a molecule’s chemical structure and its biological activity. SAR is a fundamental principle in medicinal chemistry and pharmacology, helping to clarify how structural features influence biological effects [38]. Therefore, different synthetic approaches were employed to evaluate the effect of various substituents on the three-ring system and the activity of the synthesized compounds, as brief in SI Table 2.

**Figure 2:**
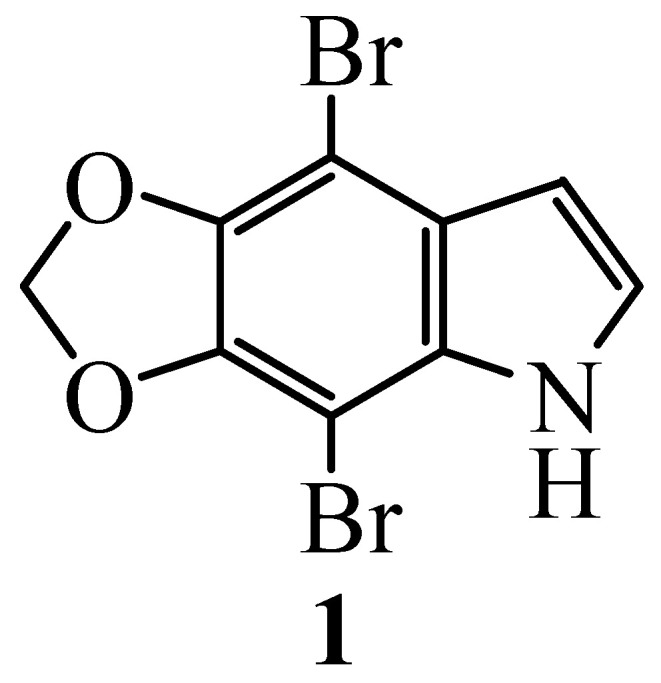
Chemical structures of amakusamine (**1**) [37].

For instance, a study by Maeyama et al., (2021) [37] explored the SAR of **1** (Figure 2) using a series of synthetic routes. Compound **1** was isolated from a *Psammocinia* sponge, whereas **1a** is its derivative. Compound **1a** (SI Scheme 1) was resynthesized from 6-nitropiperonal (**4**) via dibromination using *N*-bromosuccinimide (NBS) in conc. H_2_SO_4_. A total synthesis approach, which is related to NP isolation [39] was employed to resynthesize **1**. This implies that the proposed structure of **1** isolated from the sponge is correct, which bridges NP isolation with synthetic chemistry, as both structure validation and enabling the scale-up of the compound for further pharmaceutical purposes [39] as a SAR study. Next, 2, 3-dibromoindole (DBI, **3**) was obtained by the dibromination of 5, 6-methylenedioxyindole (**2**) using NBS in tetrahydrofuran (THF) at 0 °C under argon (SI Scheme 1) [37]. In SI Scheme 1**,** the electrophilic addition reaction afforded the vicinal dibromide (**3**). However, saturation of the indole double bond led to a reduction in inhibitory activity. Thus, the presence of two Br atoms at the 4- and 7-positions of the indole core is essential for potent activity, rather than vicinal dibromination of the double bond (Table 1). 

Thus, Maeyama et al., (2021) [37] followed a method according to Huleatt et al., (2011) [40], by reacting **4** with NBS in H_2_SO_4,_ and obtained 4, 7-dibromo-6-nitropiperonal (DBNP, **5**). Next, the Henry reaction was carried out with Al_2_O_3_ (base) in nitromethane and acetic anhydride (Ac_2_O), which formed dinitro (**6**), and finally, its reductive cyclization using Fe, SiO_2_ in acetic acid yielded **1a** (SI Scheme 1). Further, the reduction reaction of **1** indole double bond was carried out by stirring with sodium cyanoborohydride (NaBH_3_CN) in 40% acetic acid at room temp. for 23 h and then quenched with cold NaHCO_3_ solution at 0 °C, which afforded 4,8-dibromo-6,7-dihydro-5H-[1,3]dioxolo[4,5-f]indole (DH-DBDI, **7**). According to Huleatt et al., (2008) [41] the synthesis of 4,7-dibromo 5,6-dihydroxyindole derivative (DHICA, **8**) was produced by treating the 5,6-dihydroxyindole-2-carboxylic acid (SI Scheme 2) with vinylmagnesium bromide in THF [37].

Then, **S1** was used in the synthesis of **9**, and the **S2** derivative was used in the synthesis of **10** (SI Scheme 3). A 4,7-dichloro-6-nitrobenzo[d][1,3]dioxole-5-carbaldehyde (**S3**) was prepared using **4** in 35% H_2_SO_4_/trichloroisocyanuric acid (TCCA), which was used in the synthesis of 4, 7-dichloroindole (**11**). Thereafter, *N*-acetyl (**12**) was synthesised using a solution of **1** in Ac_2_O and 4-dimethylaminopyridine (DMAP) and Et_3_N at room temp. In the synthesis of **13**–**15**, compound **1** was used with 1, 2-dichloroethane (DCE), DMAP, Et_3_N, and n-butyryl chloride at room temp., and stirred for 15 h at 70 °C. To synthesize *N*-benzoyl derivative (**16**) (SI Scheme 4), compound **1** was reacted with DMAP, Et_3_N, and benzoylchloride at room temp. for 12 h and purified.

In the same study by Maeyama et al., (2021) [37] employing the method from Huleatt et al., (2011) [40], 3-formyl (**19**) was synthesised from **1** (SI Scheme 4). A solution of dimethylflouride (DMF) and phosphoryl chloride (POCl_3_) was added at 0 °C under argon and stirred for 10 min. Later, for the synthesis of the 3-methyl derivative, 4,8-dibromo-3-methyl-5,7-dihydroxyindole (DBMI, **17**) of **1**, a solution of **19** was treated in dry THF/LiAlH_4_ at 0 °C, stirred for 1 h at room temp., and purified. For the synthesis of 3-hydroxymethyl derivative (**18**), a solution of **19** in THF/MeOH was treated with NaBH_4_ at room temp. and quenched with saturated aq. NH_4_Cl solution and purified. Following, **19** was utilized in the synthesis of tryptamine derivative **20** (SI Scheme 4). 

The biological activity of **1** was screened for cytotoxicity, antimicrobial activity, inhibition of RANKL-induced osteoclast formation, and the ubiquitin-proteasome system, including proteasome activity, the E1 enzyme, the Ubc13-Uev1A interaction, the p53-Mdm2 interaction, and USP7. Of these, **1** selectively inhibited receptor activator of nuclear factor κB ligand (RANKL)-induced formation of multinucleated osteoclasts in RAW264 cells, with a half-maximal inhibitory concentration (IC_50_) value of 10.5 μM [37] via the suppression of nuclear factor of activated T-cells, cytoplasmic 1 (*Nfatc1*) expression [42]. As shown in Table 1, a study by Maeyama et al., (2021) [37] indicated that **1** inhibited the formation of (RANKL)-induced formation of multinuclear osteoclasts with an IC_50_ of 10.5 μM, and its synthetic analogue **1a** displayed equivalent efficacy (IC_50_ = 9.4 μM). The effect of bromine atoms on the indole ring of **2** and **3** had an impact on the IC_50_; **2** exhibited no inhibitory activity, whereas **3** showed 40% inhibition at 50 μM (Table 1).

Next, the effect of the double bond manifested in **7** reduced its activity to an IC_50_ value of 25.6 μM, revealing that the presence of the double bond is important for its activity, whereas the methylenedioxy group [40] replaced with two methoxy groups in **8**, further improved its activity to 6.3 µM. The indole scaffold modification in **20** (IC_50_ = 5.9 µM) with a tryptamine derivative resulted in nearly a two-fold RANKL inhibition compared to its natural product 1 (IC_50_ = 10.5 µM). Whereas the *N*-acyl derivatives of the indole scaffold bearing long-chain alkyl (**15**) and *N*-benzoyl derivative (**16**) groups show no inhibitory activity at 50 µM. In 4-monobromoindole (**9**) (IC_50_ = 16.8 µM) and 7-monobromoindole (**10**) (IC_50_ = 35.4 µM), the position of the -Br group plays a key role and shows vastly different inhibitory activity. Of the derivatives, Br (**20**, IC_50_ = 5.9 µM) substituted derivatives on the amakusamine ring exhibited eight-fold greater activity than the -Cl substituted derivative (**11**, IC_50_ = 40.0 µM) [37]. 

However, *N*-acyl (**15**) and *N*-benzoyl (**16**) modifications on the pyrrole ring of **1** resulted in a loss of activity against RANKL. Comparing the effect of bromine and chlorine atoms on inhibitory activity, compound **3** with bromines at C-2 and C-3, exhibited 40% inhibition at 50 μM, whereas 4,7-dichloroindole (**11**) with chlorines at C-4 and C-7, displayed weaker activity (IC_50_ = 40.0 μM) [37]. Thus, compound **1** was modified due to its activity against RANKL, a key signalling protein that plays a critical role in osteoclast differentiation and activation.

**Figure 3:**
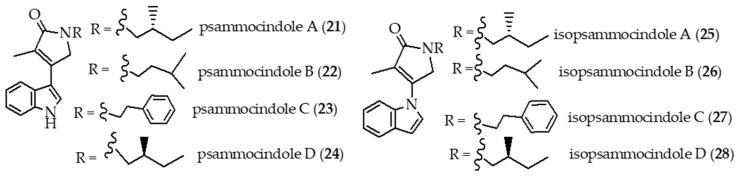
Synthetic analogues **24**–**28** derived from compounds **21**–**23** [6].

A *Psammocinia vermis*, collected by SCUBA diving from Chuja-do, South Korea, at a depth of 15–20 m [19], yielded psammocindoles A-C (**21**–**23**, Figure 3), a new class of non-brominated indole alkaloids [6]. These compounds are enantiomers of psammocindole D (**24**), and their corresponding *N*-lactam isomers, isopsammocindoles A-D (**25**–**28**, Figure 3), were also obtained through a direct synthetic route. Structurally, the psammocindoles consist of a linear arrangement of three distinct moieties: an indole, a *γ*-lactam, and an *N*-alkyl group, which are likely biosynthetically derived from the amino acids isoleucine, leucine, and phenylalanine, respectively [6].

In the pursuit of exploring the biological activity of the indole scaffold, a total synthesis of psammocindoles (**21**–**23**, Figure 3) isolated from the sponge *Psammocinia vermis* was carried out, and this synthetic effort yielded *N*-lactam isomers known as isopsammocindoles A-D (**25**–**28**, SI Scheme 5) [6]. The SAR studies of these compounds were focused on evaluating the effects on adiponectin biosynthesis, a key factor implicated in various human metabolic diseases, including obesity, type 2 diabetes, atherosclerosis, and non-alcoholic steatohepatitis [6]. Compounds **21**–**28** (10 μM) enhanced adiponectin secretion during adipogenesis in human bone marrow-derived mesenchymal stem cells (hBM-MSCs) compared to that in isobutyl methylxanthine (IBMx). Of these, **21** and **22** (Figure 3) were more effective with an EC_50_ of 9.86 and 6.20 μM, respectively, than bezafibrate (EC_50_ >10 μM), a prescribed pan-PPAR agonist, whereas **23**–**28** (Figure 3 and SI Scheme 4) were less significant (EC_50_ >10 μM) [6]. Therefore, in comparison, the natural product isolates (**21**–**22**) exhibited greater potency than their derivatives (**24**–**28**).

#### 1.3.2. Polyketides and Peptides

**Figure 4:**
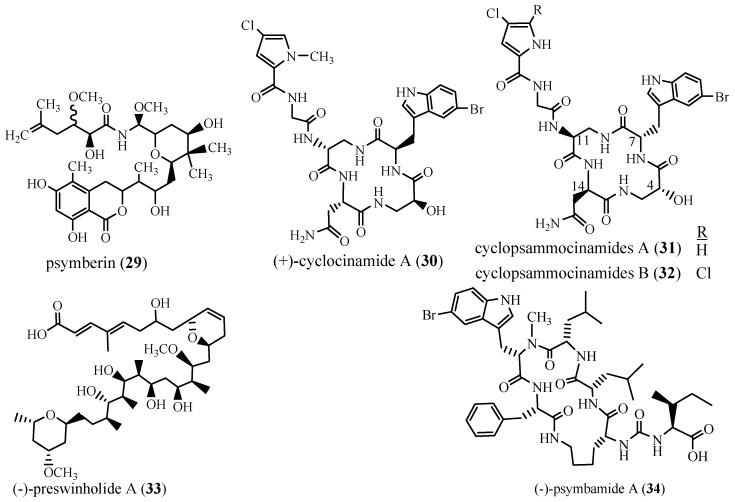
Polyketides (**29**, **33**, and **34**) and peptides (**30**, **31**, and **32**) isolated from *Psammocinia* sp. [46].

Methanolic soluble fraction of the marine sponge *Psammocinia aff. bulbosa*, collected from the waters of Papua New Guinea at a depth of 9–18 m [16], yielded the cytotoxic polyketide (+)-psymberin or irciniastatin A (**29**). Compound **29** (Figure 4) was also reisolated from the recollection of *P. aff. bulbosa* in Papua New Guinea, which showed differences in biological activity. It was further tested against six distinct colorectal cancer (CRC) patient-derived organoid models, including CRC057, CRC401, CRC403, CRC119, CRC16-159, CRC240, CRC247, CRC19-178, and exhibited IC_50_’s < 25 nM [43].

For comparison, *Psammocinia* collected from Papua New Guinea, prepared between 1990 and 2001 from an unidentified depth, resulted in **29**, which was further tested in vitro against various cancer cell lines. It exhibited an LC_50_ sensitivity of >2.5 × 10^−5^ M against leukemia cell lines (CCRF-CEM, HL-60 (TB), K-562, MOLT-4, RPMI-8226, and SR). However, it showed higher potencies against the following breast cancer cell lines: MDA-MB-435 < 2.5 × 10^−9^ M, NCI/ADR-RES (1.9 × 10^−5^ M), and T-47D (1.36 × 10^−5^ M), while it was less potent against MCF7 and HS578T, LC_50_’s of >2.5 × 10^−5^ M [44]. When tested against the following melanoma cell lines, LOX IMVI, UACC-257, and SK-MEL-2, compound **29** demonstrated LC_50_ > 2.5 × 10^−5^ M, whereas melanoma cell lines: MALME-3M, SK-MEL-5, and SK-MEL-28were more sensitive with LC_50_ of <2.5 × 10^−9^ M and 1.41 × 10^−5^ M against SK-MEL-28, respectively. Further, it was tested against various colon cancer cell lines with LC_50_ > 2.5 × 10^−5^ M against (HT29 and SW-620), LC_50_ < 2.5 × 10^−9^ M against HCT-116, and 3.76 × 10^−7^ M against HCC-2998 [44]. 

In addition, a cyclic peptide, (+)-cyclocinamide A (**30**), was also isolated in 1997 from a *Psammocinia* sp. collected in Papua New Guinea [42,45]. Compound **30** (Figure 4) was tested against a single mouse with early-stage M-16 (mammary adenocarcinoma no. 16) and administered a total dose of 20 mg/kg; however, no antitumor activity or toxicity was observed [45]. Compound **30** was also reported from *Psammocinia aff. bulbosa* [15,16]. The dichloromethane extract of *Psammocinia aff. bulbosa* collected in Milne Bay, Papua New Guinea, at a depth of 9–18 m produced (-)-preswinholide A (**33**), and polypeptide (-)-psymbamide A (**34**), unique to the species, were not tested for biological activity in the original isolation [15,16].

Later, in 2022, two structurally related cyclic peptides, cyclopsammocinamides A (**31**) and B (**32**), were obtained from an ethanol extract of *Psammocinia* sp. collected by SCUBA diving at a depth of 10 m from North Sulawesi, Indonesia. Compounds **31** and **32** (Figure 4) are enantiomers of **30**, which was reisolated from *Psammocinia* sp.; however, neither **31** nor **32** exhibited cytotoxic activity against HCT-116 colon cancer cells, whereas **30** showed 6% viability at 50 µg/mL against HCT-116 cancer cells [46].

The biosynthesis route of **29** [47,48] indicated in (SI Scheme 6) has a complex structure, featuring multiple stereocenters (5*S*, 8*S*, 9*S*, 11*R*, 13*R*, 15*S*, 16*R*, 17*R*), which makes **29** an attractive target for total synthesis [49]. The Barbier reaction, a carbon–carbon bond formation, has been effectively utilized in the synthesis of structurally complex NPs, such as compound **29** [50]. In 2019, Yu et al., [48] achieved a convergent, stereo-controlled total synthesis of **29** starting from the known chiral aldehyde (*R*)-2-(2,2-diethyl-1,3-dioxolan-4-yl)acetaldehyde (DDA, **35**), employing a Barbier reaction under ultrasonic irradiation [48]. In this step, **35** was treated with prenyl bromide (**36**) and freshly activated Zn powder under ultrasound, yielding a mixture of intermediates (**37**) and (**38**) [48,49].

This reaction enables the formation of primary, secondary, or tertiary alcohols by treating a carbonyl compound with an alkyl halide in the presence of a metal such as Mg, Zn, Al, In, or their salts [49]. Compounds **35**–**38** were used as starting materials for the biosynthetic route of **29** and were therefore not investigated for their biological activity.

#### 1.3.3. Terpenoids

##### Furanosesterterpene (C25): Furan-Containing Terpenoids

**Figure 5:**
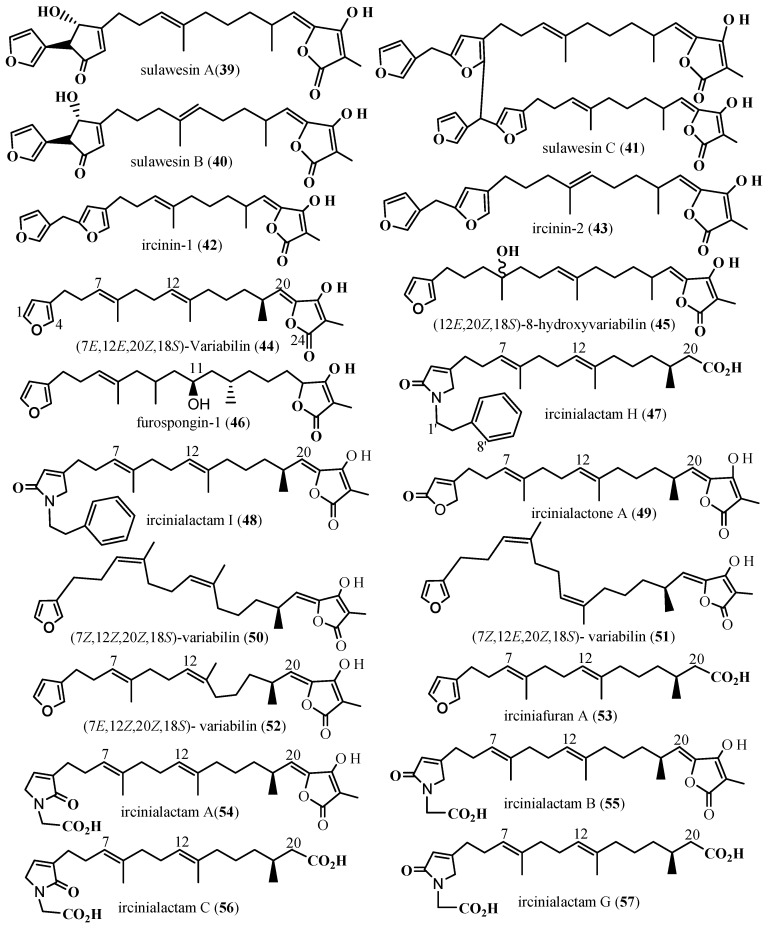
Furanosesterterpenes [51,52,55].

Ethanol extract of *Psammocinia* sp., collected in North Sulawesi, Indonesia, yielded three new furanosesterterpene tetronic acids, including sulawesins A-C (**39**–**41**), along with the known ircinin-1 (**42**) and ircinin-2 (**43**). Compounds **42** and **43** were enantiomeric mixtures, whereas sulawesin A (**39**) and sulawesin B (**40**) were identified as diastereomeric mixtures (Figure 5) [51]. Compounds **39**–**43** exhibited enzyme inhibitory activity against ubiquitin-specific protease 7 (USP7), a deubiquitinating enzyme that has emerged as a promising target in cancer therapy. These compounds inhibited USP7 with IC_50_’s from 2.7 to 4.6 μM [51]. Marine sponges collected from Indonesia, including *Ircinia* sp. and *Spongia* sp., yielded furanoterpene-based inhibitors of protein tyrosine phosphatase 1B (PTP1B). Compounds (7*E*,12*E*,20*Z*,18*S*)-variabilin (**44**) and (12*E*,20*Z*,18*S*)-8-hydroxyvariabilin (**45**), isolated from *Ircinia* sp., along with furospongin-1 (**46**) from *Spongia* sp., showed dose-dependent PTP1B inhibition with IC_50_ of 1.5, 7.1, and 9.9 μM, respectively. Compound **44** was about five times more potent than **45**, suggesting that the -OH group at the C-8 position in **45** may negatively affect its PTP1B inhibitory activity (Figure 5) [52]. 

A methanol extract of a sponge identified as Sarcotragus sp., collected from Kaikoura, New Zealand, led to the purification of the furanosesterterpene tetronic acid variabilin (**44**). Compound 44, initially discovered in *Ircinia* variabilis, is a major constituent in the genera *Ircinia*, *Psammocinia*, and Sarcotragus of the New Zealand sponge collections. It has been recognized as the major bioactive compound in extracts from *Sarcotragus* species (order Dictyoceratida) [52,53].

In addition, the inhibitory effects of 44 and 45 from *Ircinia* sp. and 46 from *Spongia* sp. on various protein tyrosine phosphatases (PTPs), including T-cell PTP (TCPTP), which shares 72% sequence identity with PTP1B [54], were evaluated using an in vitro enzyme assay. Compounds **44** and **45** showed almost twofold greater potency against TCPTP compared to PTP1B, with IC_50_ of 0.8 μM vs. 1.5 μM for 44, and 3.7 μM vs. 7.1 μM for **45**, respectively. In contrast, **46** inhibited TCPTP and PTP1B to a similar extent (IC_50_ = 9.6 μM vs. 9.9 μM), respectively. Moreover, 44 displayed comparable inhibition of PTP1B and TCPTP, while their activity against vaccinia H1-related (VHR) tyrosine phosphatase was four times weaker than that against PTP1B. Compounds **44**–**46** (Figure 5) did not exhibit cytotoxicity against two human cancer cell lines, hepatoma (Huh-7) and bladder carcinoma (EJ-1), with an IC_50_ of 50 μM after 72 h. Compound **44** did not enhance the phosphorylation level of serine/threonine kinase referred to as the Albert Kennedy trust (AKT), a key downstream effector of the cascade, in Huh-7 cells [15,52]. 

A study by Prasad et al., (2018) [55] on *Psammocinia* sp., collected in 1988 via SCUBA diving off at 15 m from the coast of New South Wales, Australia resulted in ircinialactams H (**47**) and I (**48**), ircinialactone A (**49**), four variabilin analogies including compound 44, (7Z,12Z,20Z,18S)-variabilin (**50**), (7Z,12E,20Z,18S)- variabilin (**51**), and (7Z,12E,20Z,18S)-variabilin (**52**) and irciniafuran A (**53**). Further fractionation afforded ircinialactams A (**54**), B (**55**), C (**56**), and G (**57**) (Figure 5). These compounds were not evaluated for their biological activities during the study. This conveys that the genus *Psammocinia* is recognized for producing a variety of biologically active furanosesterterpenes (furan-containing sesterterpenoid analogues), including variabilins [56], strobilinins [57], ircinins [58], sarcotins [59], and psammocinins [60]. Notably, these compounds exhibited different absolute configurations at the C-18 position [51]. 

A *Psammocinia* sp., collected by SCUBA diving off Ulleung Island, South Korea, at a depth of 20 m, in October 2001, yielded sesterterpenes including psammocinin A_1_ (58), psammocinin A_2_ (**58**), psammocinin B (**60**), palinurin (**61**), and isopalinurin (**62**). Additionally, a mixture of (8E,13Z,18R,20Z)-strobilinin (**63**) and (7E,13Z,18R,20Z)-felixinin (**64**), as well as a mixture of (8Z,13Z,18R,20Z)-strobilinin (**65**) and (7Z,13Z,18R, 20Z)-felixinin (66), furanosesterterpenes were also isolated [14,60].

**Figure 6:**
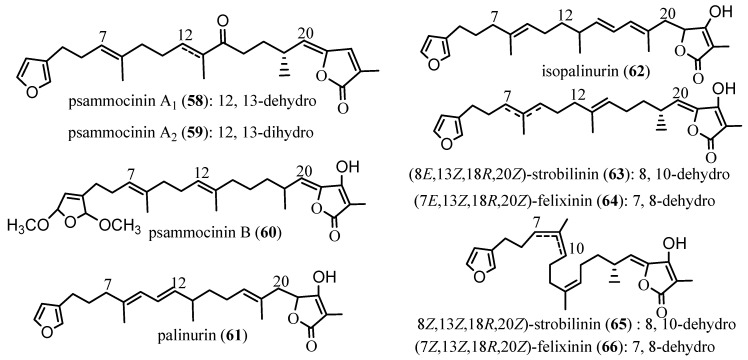
Sesterterpenes (**58**–**62**) and Furanosesterterpenes analogues (**63**–**66**) [14,60].

The cytotoxicity of compounds **44** and **58**–**66** was evaluated against five human solid tumour cell lines, including A549 (lung), SK-OV-3 (ovarian), SK-MEL-2 (melanoma), XF498 (central nervous system), and HCT15 (colon). Compound **61** exhibited the most potent activity across all cell lines, with effective dose (ED_50_) values ranging from 4.4 to 10.2 µM [14]. Compounds **59** and **60** also showed moderate cytotoxicity (ED_50_ = 4.8 to 19.2 µM), while **58**, **62**, **65**, and **66** (Figure 6) were largely inactive (ED_50_ >30 µM) against several cell lines. Notably, **63** and **64** demonstrated similar and consistent activity across the panel, with ED_50_ between 5.8 and 11.0 µM against SK-MEL-2 cell lines and **44** with ED_50_ between 18.6 and 24.1 µM (SI Table 2) [14,15,60].

##### Sesterterpenes (C25 Terpenoids)

An extract of *Psammocinia* sp. using 50% MeOH in DCM, collected from South Korea, yielded three novel tetracyclic sesterterpenoids (C_25_ terpenes, scalarane-type) along with four known analogues [5]. Fractionation of the extract using a Sephadex LH-20 resulted in the isolation of **67**–**73**. Three new compounds were identified as 12-deacetoxy-23-hydroxyscalaradial (**67**), 12-dehydroxy-23-hydroxyhyrtiolide (**68**), and 12-O-acetyl-16-deacetoxy-23-acetoxyscalarafuran (**69**). The four known compounds include: 12-deacetoxy-23-hydroxyheteronemin (**70**), 12-deacetoxy-23-acetoxy-19-O-acetylscalarin (**71**), 12-deacetoxy-23-O-acetoxyheteronemin (**72**), and 12-deacetoxyscalaradial (**73**). All compounds are cytotoxic with an in vitro activity against human cancer cell lines, including human renal cancer cell lines (A498, ACHN), pancreatic cancer cell lines (MIA-paca, PANC-1), and a noncancerous monkey cell line (CV-1), with IC_50_ ranging from 0.4 to >50 μM [5] (SI Table 2). 

**Figure 7:**
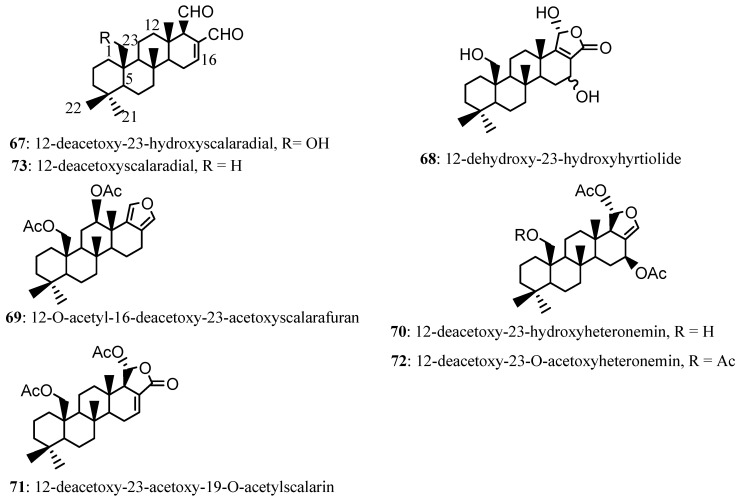
Scalarane sesterterpenes (**67**–**73**) [5,61,62].

Compounds **69**–**71** (Figure 7) were also isolated from the MeOH extract of *Smenospongia* sp. collected by SCUBA diving at 15–20 m depth off the shore of Gagu-Do (Island), Southwestern Korea in the year 2000. Compounds **70** and **71** exhibited cytotoxic activity against the human leukemia cell line K562 with LC_50_’s of 0.02 and 4.9 µg/mL, respectively [61]. Four years later, Song et al., (2008) [62] reported nearly the same LC_50_ values of 0.13 and 4.9 µg/mL for **70** and **71**, respectively, isolated from *Smenospongia* sp. collected by SCUBA diving at depths of 25–30 m off Soheuksan Island, Korea in 2006. Scalarane sesterterpenes, including **69**–**71** (Figure 7), exhibited various bioactivities [61]. For instance, these compounds were reported to have moderate antibacterial and weak inhibitory activity against isocitrate lyase, with an IC_50_ > 100 µg/mL [62]. Compound **70**, isolated from *Smenospongia* sp., exhibited significant activity against *Salmonella typhimurium* (ATCC 14028) and *Bacillus subtilis* (ATCC 6633) with minimum inhibitory concentration (MICs) of 6.25 and 0.78 µg/mL, respectively. Compound **71**, identified from *Smenospongia* sp., was active against *B. subtilis* (MIC = 3.12 µg/mL) but exhibited no antibacterial activity with MIC >100 µg/mL against *S. aureus*, *M. leuteus*, *S. typhimurium*, *P. Vulgaris*, and *E. coli* [62] (SI Table 2).

**Scheme 1:**
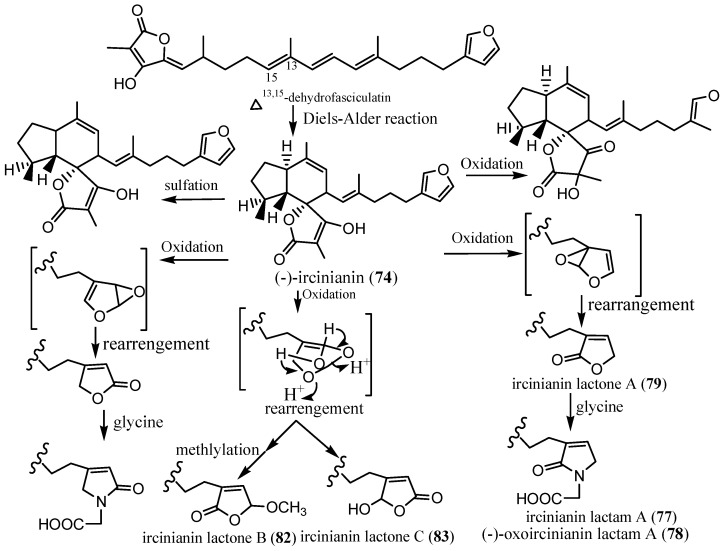
A biosynthetic route to ircinianin-type sesterterpenoids [53].

An ethanol extract of three *Psammocinia* sp. (CMB-01008, CMB-02858, and CMB-03344) collected from the Southern Australian Sea at an unidentified depth led to the isolation of sesterterpenes, including (-)-ircinianin (**74**) and (-)-ircinianin sulphate (**75**) isolated from CMB-01008. In addition, other biosynthetically related metabolites (Scheme 1) were also isolated, including (-)-ircinianin lactam A (**76**), identified from CMB-03344; (-) ircinianin lactam A sulphate (**77**) from CMB-01008; (-)-oxoircinianin (**78**) was reported from CMB-03344; (-)-oxoircinianin lactam A (**79**), and (-)-ircinianin lactone A (**80**), both isolated from the CMB-02858 sample ID [63]. Acetylation of **74** in a synthetic transformation led to (-)-ircinianin acetate (**81**) [53].

**Figure 8:**
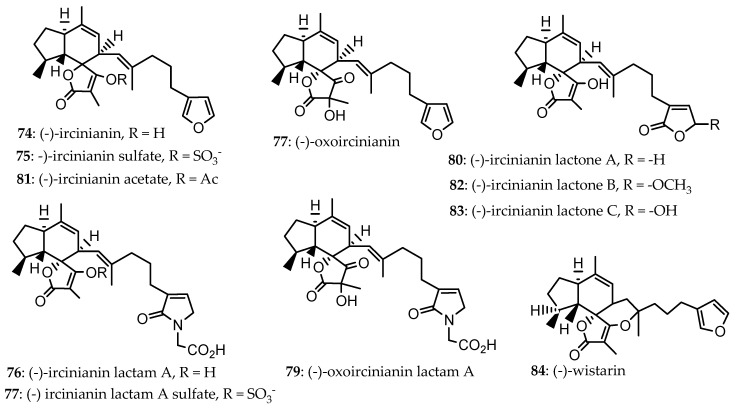
*Ircinianins* type isolated from *Psammocinia* sp. and its derivative **84** [63] from *I. wistarii* [53].

Glycine-gated chloride channel receptors (GlyRs) play a crucial role in coordinating inhibitory neurotransmission in the spinal cord, brainstem, and retina. In the biological activity evaluation using whole cell patch-clamp electrophysiology of compounds **74**–**81** (Figure 8), they were screened against human α1 and α3 GlyRs stably expressed in HEK293 cells through automated planar chip whole cell patch-clamp. Of these, **76** was an exceptionally potent and selective α3 GlyR potentiator, whereas **79** was selective against α1 GlyR potentiator (110±8% at 100 μM) [63]. Compound **75** was an antagonist for both α1 and α3 with IC_50_ = 38.4 ± 2.8 μM and 3.2 ±2.1 μM, respectively. Compounds **74** and **77** were modest against α1 GlyR potentiators (80 ± 10% and 70 ± 7%, at 100 μM, respectively), and **76**, **78**, **80**, and **81** showed no effect [63].

Later, in 2022, a 1:1 CHCl_3_:MeOH extract of *Ircinia wistarii* collected by SCUBA diving from the Great Barrier Reef, Australia, at a depth of 20 m, resulted in two new ircinianin-type sesterterpenoids, including ircinianin lactone B (**82**) and ircinianin lactone C (**83**), which were isolated along with five previously reported members of the ircinianin family (**74**, **76**–**79**). Compounds **82** and **83** (Figure 8) represent novel ircinianin terpenoids featuring a distinct oxidation pattern [53].

In a broad biological screening, compound **74** (Figure 8) isolated from *I. wistarii* exhibited a moderate antiprotozoal activity against *Plasmodium falciparum* (IC_50_ = 25.4 µM) and *Leishmania donovani* (IC_50_ = 16.6 µM). The antiviral potential of **74** was tested against the human cytomegalovirus (HCMV) and the severe acute respiratory syndrome coronavirus 2 (SARS-CoV-2); however, no inhibition was seen at a single dose screen of 1 and 10 µM, respectively. Further, **74** was evaluated against female adult worms, *Litomosoide sigmodontis*, where no inhibition of worm motility was observed at 0.1, 1, and 10 µM. Compound **74** was also evaluated against the ESKAPE panel (*E. faecium* BM4147-1, *S. aureus* ATCC29213, *K. pneumoniae* ATCC12657, *A. baumannii* 09987, *P. aeruginosa* ATCC27853, *E. aerogenes* ATCC13048, *E. coli* ATCC25922, *B. subtilis* 168) and a mycobacterial strain (*M. smegmatis* mc^2^ 155); however, it showed no activity with MIC > 32 µg/mL. Compound **74** exhibited no cytotoxic activity in the National Cancer Institute’s (NCI) 60 human tumour cell-line screening and was also inactive against rat skeletal myoblasts (L6) (IC_50_ = 59.5 µg/mL) and HeLa cells (IC_50_ > 64 µg/mL) in cell line assays. Compounds **76**–**83** isolated from *I. wistarii* and **84**, the derivative of **74**, were not bioassayed [53]. However, **74** isolated from (CMB-01008, CMB-03344), and *I. wistarii* showed broad-spectrum biological activity, including modulation of human α1 and α3 glycine-gated chloride channel receptors, antiprotozoal, and antiviral activities, while showing no cytotoxicity in mammalian cell lines. The synthesis of ircinianins (Scheme 1) was derived from a linear triene precursor via an intramolecular Diels-Alder reaction through enzymatic catalysis [64]. A crucial step in the biosynthesis routes of ircinianins is hypothesized to include partial oxidation, which often leads to intramolecular cyclization and rearrangement processes. These changes result in the formation of five-membered heterocycle derivatives, including furans and lactones [53].

##### Meroterpenoids or Terpenoid-Polyketide Hybrids

**Scheme 2:**
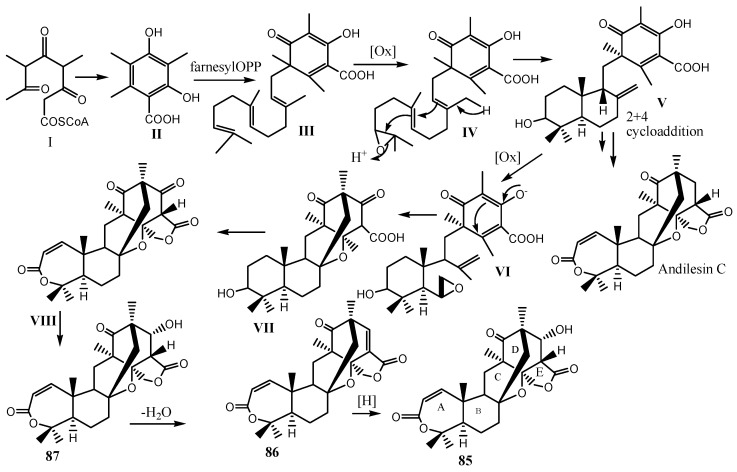
Biosynthesis route to **85**–**87**, closely related to that of andilesins and andibenins [65,66].

A study of fungi associated with the marine sponge *Psammocinia* sp., collected from the Mediterranean Sea via SCUBA diving off the coast of Sdot-Yam, Israel, at a depth of 20 m, led to the isolation of three new meroterpenoids: insuetolides A-C (**85**–**87,** Scheme 2), along with four drimane-type sesquiterpenes (**88**–**91**). 

**Figure 9:**
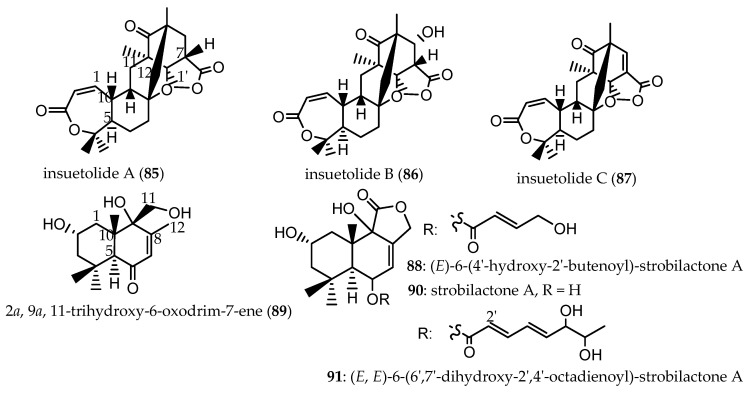
Meroterpenoids (**85**–**87**) and drimane-type (**88**–**91**) isolated from the marine-derived fungus *A. insuetus*, associated with *Psammocinia* sp. [42,65].

These included one newly identified (*E*)-6-(4′-hydroxy-2′-butenoyl)-strobilactone A (**88**), and three known drimane sesquiterpenes: 2α, 9α, 11-trihydroxy-6-oxodrim-7-ene (**89**), strobilactone A (**90**), and (*E, E*)-6-(6′,7′-dihydroxy-2′,4′-octadienoyl)-strobilactone A (**91**) which were isolated from the marine-derived fungus *Aspergillus insuetus* (OY-207) [42,65]. Compounds **85**–**91** (Figure 9) were obtained as transparent glassy solids. Compounds **85**, **90**, and **91** exhibited MIC values of 140, 242, and 162 μM, respectively, against the fungus *Neurospora crassa*. In cytotoxicity assays against the MOLT-4 human leukemia cell line at 50 μg/mL, **87**, **88**, and **91** (Figure 9) inhibited cell proliferation by 51%, 55%, and 72%, respectively, whereas **85** and **90** showed no significant activity at the same dosage [65].

Meroterpenoids are primarily isolated from fungi and marine organisms, although bacteria and higher plants can also produce these hybrid biosynthetic products. Among fungi, polyketide–terpenoid-derived meroterpenoids represent the largest class, with those derived from orsellinic acid or its mono- and di-methyl derivatives being the most reported, particularly from *Penicillium* and *Aspergillus* species [66]. Meroterpenoids originating from 3,5-dimethylorsellinic acid (II) are formed via a common intermediate, generated by the alkylation of 3,5-dimethylorsellinic acid with farnesyl diphosphate (III). This precursor gives rise to a wide structural diversity of secondary metabolites through multiple biosynthetic modifications, including multi-step oxidation, as observed in the andibenins, backbone rearrangements, as in the andilesins, anditomins, and tropolactones, and backbone fragmentation, shown by the austin and terretonin families [65,66]. 

Insuetolides A-C (**85–87**) feature a novel meroterpenoid skeleton closely related to the andilesins and andibenins (Scheme 2). The key difference lies in ring C, which is a cyclopentane in the andilesins and andibenins, but a perhydropyran ring in the insuetolides, suggesting an additional oxidation step occurs before the coupling of C-12 to the tetraketide moiety (VII). Based on this, a proposed biosynthetic pathway (Scheme 2) begins with the formation of a drimane-type sesquiterpene linked through C-11 to C-30 of the orsellinic acid unit. The 8, 12-terminal double bond of this intermediate undergoes epoxidation, which subsequently participates in a phenol-oxidation-type coupling with C-12 and the C-8 oxy-radical. The remaining steps of the biosynthesis mirror those established for the andilesins, ultimately generating the structurally unique **85**–**87** [65,66].

### 1.4. Other Miscellaneous

A 1:1 (*v*/*v*) DCM: MeOH extract of *Psammocinia* sp., collected off the Persian Gulf, Iran, at a depth of 10-12 m, was evaluated for its antimicrobial and antibiofilm activities against six bacterial strains: *Pseudomonas aeruginosa*, *Acinetobacter baumannii*, *Klebsiella pneumoniae*, *Escherichia coli*, *Staphylococcus aureus*, and *Bacillus cereus.* Chemical profiling of the extract was shown using Gas Chromatography-Mass Spectrometry (GC-MS), which revealed the presence of carboxylic acid esters, valtrate, phenolic compounds, and benzoic acid derivatives as major constituents [67]. Antimicrobial testing using disk diffusion and agar well diffusion methods demonstrated zones of inhibition for all tested strains except *K. pneumoniae*. The *Psammocinia* sp. extract exhibited MICs ranging from 10 to 20 mg/mL and minimum bactericidal concentrations (MBCs) between 20 and 80 mg/mL. Despite the absence of a zone of inhibition, the extract showed strong inhibition of biofilm formation when tested at three dilutions of 12, 6.25, and 3.12 mg/mL, with inhibition rates of 90.32% against *K. pneumoniae* biofilms and 90.86% against *P. aeruginosa* biofilms [67]. 

### 1.5. Conclusion and Future Perspectives

This review highlighted that the bioprospecting of the marine sponge *Psammocinia* sp. led to the isolation and characterization of various classes of compounds from different geographical locations, targeting diverse areas of drug discovery. NPs derived from various organisms display remarkable structural diversity and exhibit a wide range of biological activities, including antiplasmodial, anti-inflammatory, anticancer, and antimicrobial effects. To date, numerous compounds have been sourced from natural sources and utilized in drug discovery and development. This review emphasized the limited pharmacological profiling, lack of *in vivo* antibacterial activity, and in vitro enzyme-inhibitory studies among compounds identified and derivatised from *Psammocinia* sp. chemical investigations. This implies that NPs reported from *Psammocinia* sp. have received little attention for their potential as enzyme inhibitors. Although the marine sponge *Psammocinia* sp. has been chemically investigated in identifying bioactive NPs, targeting the above-mentioned specific enzymes and other targets in the drug discovery remains largely unexplored. In this review, some structurally similar compounds reported from related sponge genera displayed different biological activities, a variation likely influenced by the chemical biogeography of these organisms. Moreover, *Psammocinia* sp. lacks the correct morphological identification, which recent morphological analysis techniques could tackle. 

## Figures and Tables

**Table 1:** The SAR study of **1** isolated from *Psammocinia* sp. and its derivatives against RANKL [37].

Chemical Structure	Inhibition (IC_50_)	Chemical Structure	Inhibition (IC_50_)
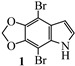	10.5 µM	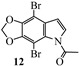	11.7 µM
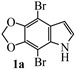	9.4 µM	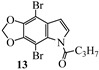	7.9 µM
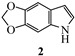	No inhibition	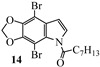	11.1 µM
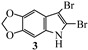	40% inhibition	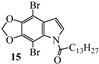	No inhibition
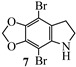	25.6 µM	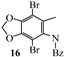	No inhibition
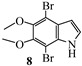	6.3 µM	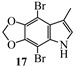	8.1 µM
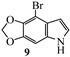	16.8 µM	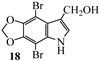	10.0 µM
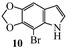	35.4 µM	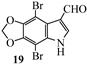	13.9 µM
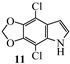	40.0 µM	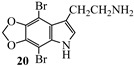	5.9 µM

## Data Availability

All data supporting the findings of this review are available within the cited literature. No new experimental data were generated in this manuscript.

## Supplementary Materials

The following supporting information can be downloaded at: https://www.mdpi.com/article/10.3390/md24040132/s1, SI Scheme 1: Resynthesis of NP amakusamine (**1**) via other derivatives (**4**–**6**); SI Scheme 2: Synthesis of derivative (**8**) from **S4**; SI Scheme 3: Synthesis of derivatives (**9**–**11**) from compound **1**’s commercial analogy (**4**); SI Scheme 4: Synthesis of derivatives (**12**–**20**) from the NP amakusamine (**1**); SI Scheme 5: Total Synthesis of psammocindoles (**24**–**28**); SI Scheme 6: Biosynthetic route of psymberin (**29**) adopted from [49]; SI Table 1: Summary of *Psammocinia* species documented to date [68]; SI Table 2: Summary of Secondary metabolites reported from *Psammocinia* sp. and their biological activity [69].

## Abbreviations

The following abbreviations are used in this manuscript:

DNA	Deoxyribonucleic Acid
ED_50_	Effective Dose 50%
IC_50_	Half-Maximal Inhibitory Concentration
LC_50_	Lethal Concentration 50%
LD_50_	Lethal Dose 50%
NBS	N-bromosuccinimide
THF	Tetrahydrofuran
NPs	Natural Products
PTP1B	Protein Tyrosine Phosphatase 1B
PTP	Protein tyrosine phosphatase
DCM	Dichloromethane
GlyRs	Glycine-Gated Chloride Channel Receptors
MIC	Minimum Inhibitory Concentration
MBC	Minimum bactericidal concentration
RANKL	Receptor Activator of Nuclear Factor-κB Ligand
SAR	Structure-Activity Relationship
SI	Supplementary Information
Sp.	Species
USP7	Ubiquitin-Specific Protease 7
WPD	World Porifera Database
μM	Micromolar
