# Supplementary material for: Secondary Metabolites Isolated from the Genus Psammocinia Sponges: Mapping Their Chemistry and Biological Activities
Source: Mar Drugs. 2026 Apr 1;24(4):132. doi: 10.3390/md24040132 (PMC13117654; doi:10.3390/md24040132)
Supplement: Supplementary file 1 [file marinedrugs-24-00132-s001.zip › marinedrugs-4183076-supplementary.pdf]

marine drugs

MDPI

Supplementary Information Scheme S1. Resynthesis of NP amakusamine (**1**) via other derivatives (**4-6**)[37,40].

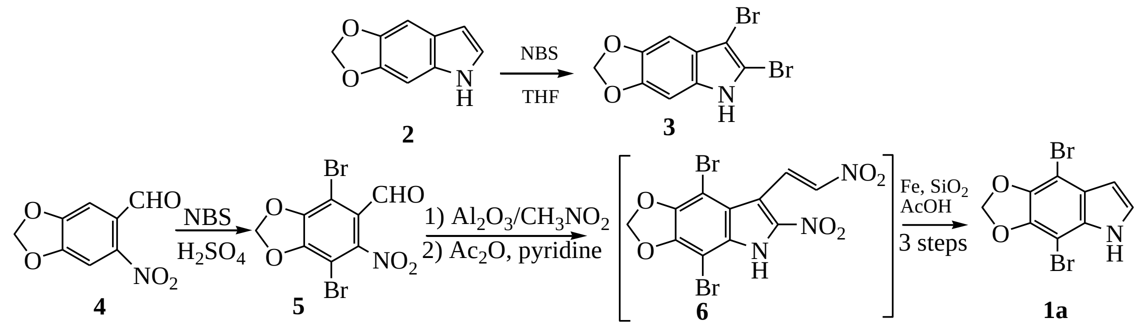

Supplementary Information Scheme S2. Synthesis of derivative (**8**) from **S4** [37,41].

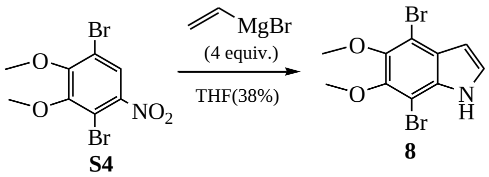

Supplementary Information Scheme S3. Synthesis of derivatives (**9-11**) from compound **1**'s commercial analogy (**4**) [37].

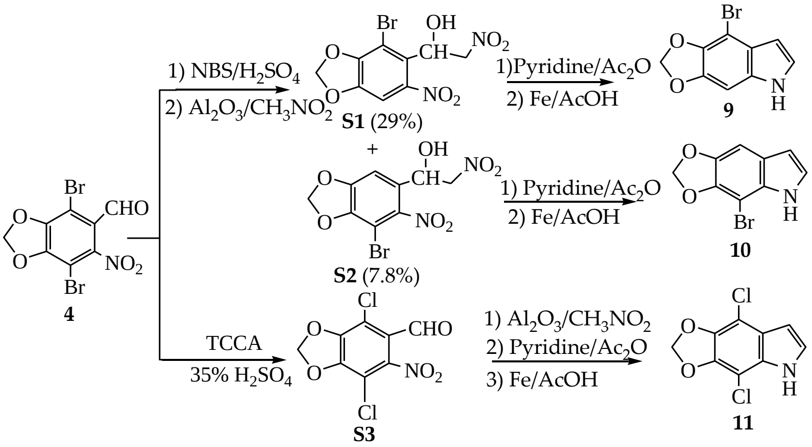

Supplementary Information Scheme S4. Synthesis of derivatives (**12-20**) from the NP amakusamine (**1**) [37].

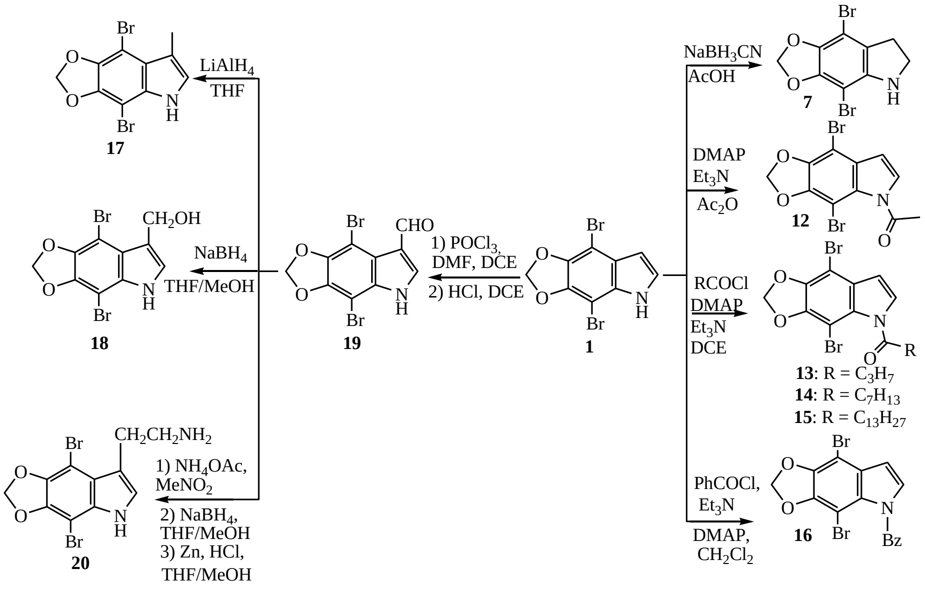

Supplementary Information Scheme S5. Total Synthesis of psammocindoles (**24**-**28**)[6].

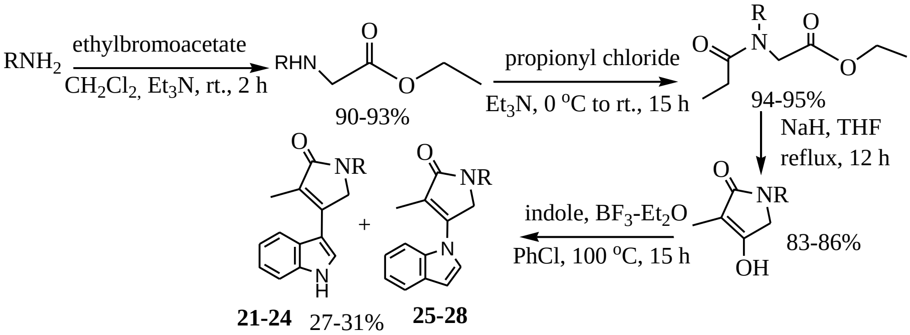

Supplementary information Scheme 6. Biosynthetic route of psymberin (**29**) adopted from [49]**.**

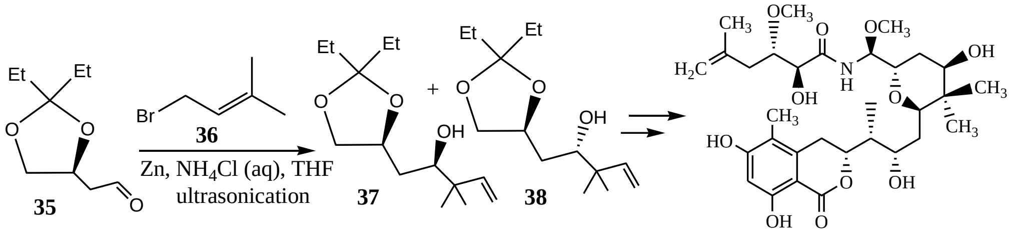

marine drugs

MDPI

Supplementary Information Table S1. Summary of *Psammocinia* species documented to date**.**

#	Scientific Name	Author and Year	Country of Origin	Ref.
1	*P. arenosa*	Lendenfeld, 1888	Australia	[31]
2	*P. halmiformis*	Lendenfeld, 1888	Australia	[31]
3	*P. vesiculifera*	Poléjaeff, 1884	Australia	[31]
4	*P. compacta*	Poléjaeff, 1884	Brazil	[17]
5	*P. bulbosa*	Bergquist, 1995	New Caledonia, Papua New Guinea	[31]
6	*Psammocinia sp.*	Lendenfeld, 1889	New Zealand, South Korea	[68]
7	*P.beresfordae*	Cook and Bergquist, 1996	New Zealand	[31]
8	*P. hawere*	Cook and Bergquist, 1996	New Zealand	[31]
9	*P. verrucosa*	Cook and Bergquist, 1996	New Zealand	[31]
10	*P. amodes*	Cook and Bergquist, 1998	New Zealand	[31]
11	*P. charadrodes*	Cook and Bergquist, 1998	New Zealand	[31]
12	*P. hirsuta*	Cook and Bergquist, 1998	New Zealand	[31]
13	*P. maorimotu*	Cook and Bergquist, 1998	New Zealand	[31]
14	*P. papillata*	Cook and Bergquist, 1998	New Zealand	[31]
15	*P. perforodorsa*	Cook and Bergquist, 1998	New Zealand	[31]
16	*P. samyangensis*	Sim and Lee, 1998	South Korea	[31]
17	*P. wandoensis*	Sim and Lee, 1998	South Korea	[31]
18	*P. jejuensis*	Sim, 1998	South Korea	[31]
19	*P. mammiformis*	Sim, 1998	South Korea	[31]
20	*P. mosulpia*	Sim, 1998	South Korea	[31]
21	*P. lobatus*	Sim and Lee, 2002	South Korea	[31]
22	*P. bergquistae*	Sim and Lee, 2001	South Korea, China	[31]
23	*P. gageoensis*	Sim and Lee, 2001	South Korea, China	[31]
24	*P. rubra*	Sim and Lee, 2002	South Korea, East China Sea	[31]
25	*P. conulosa*	Lee and Sim, 2004	South Korea, East China Sea	[31]
26	*P. massa*	Kim, Lee, and Sim, 2018	South Korea, Yellow Sea	[19]
27	*P. rana*	Kim, Lee, and Sim, 2018	South Korea, Yellow Sea	[19]
28	*P. vermis*	Kim, Lee, and Sim, 2018	South Korea, East China Sea	[19]
29	*P. chupoensis*	Sim, Lee, and Kim, 2017	South Korea, East China Sea	[18]

30	*P. ulleungensis*	Lee and Sim, 2004	East China Sea	[31]
31	*P. alba*	Calcinai *et al.*, 2017	Australia, Indonesia, South Africa	[20]
32	*P. samaaii*	Van Soest, 2024	South Africa	[18]
33	*P. aspera*	Sim, Lee, and Kim, 2017	East China Sea	[18]
34	*P. foraminis*	Sim, Lee, and Kim, 2017	East China Sea	[18]
35	*P. hwasunensis*	Sim, Lee, and Kim, 2017	East China Sea	[18]
36	*P. palma*	Sim, Lee, and Kim, 2017	East China Sea	[18]
37	*P. scopulus*	Sim, Lee, and Kim, 2017	East China Sea	[18]
38	*P. morum*	Sim, Lee, and Kim, 2017	Japan, East Sea	[20]

marine drugs

MDPI

Supplementary Information Table S2. Summary of Secondary metabolites reported from *Psammocinia sp*. and their biological activity.

**Origin (Depth)**	**Species**	**Name of Compounds**	**Classes**	**Concentration** (in Dose)	**Target activity**	**Ref.**
Japan, 5 m	*Psammocinia* sp.	amakusamine (**1**)	Alkaloid	IC_{50} 10.5 μM*	RANKL	[37]
		amakusamine (**1a**)		IC_{50} 9.4 μM*	RANKL	
		5,6-MDI(**2**)		Not assayed	-	
		DBI (**3**)		40% inhibition**	RANKL	
		6-nitropiperonal (**4**)		Not assayed	-	
		DBNP (**5**)		Not assayed	-	
		dinitro (**6**)		Not assayed	-	
		DH-DBDI (**7**)		IC_{50} 25.6 μM*	RANKL	
		7,9-DBr-DDFI (**8**)		IC_{50} 6.3 μM*	RANKL	
		4-monobromoindole (**9**)		IC_{50} 16.8 μM*	RANKL	
		7-monobromoindole (**10**)		IC_{50} 35.4 μM*	RANKL	
		4,7-dichloroindole (**11**)		IC_{50} 40.0 μM*	RANKL	
		N-acetyl compound 1 (**12**)		IC_{50} 11.7 μM*	RANKL	[37]
		Dibromo derivative (**13**)		IC_{50} 7.9 μM*	RANKL	
		Dibromo derivative (**14**)		IC_{50} 11.1 μM*	RANKL	
		N-acyl derivative (**15**)		Not assayed	-	
		N-benzoyl derivative (**16**)		Not assayed	-	
		DBMI(**17**)		IC_{50} 8.1 μM*	RANKL	
		3-hydroxymethyl compound 19 (**18**)		IC_{50} 10.0 μM*	RANKL	
		3-formyl derivative compound 1 (**19**)		IC_{50} 13.9 μM*	RANKL	
		tryptamine derivative of compound 19 (**20**)		IC_{50} 5.9 μM*	RANKL	
South Korea, 15-20 m	*P. vermis*	Psammocindole A (**21**)		EC_{50} 9.86 μM*		[6]
	P. vermis	Psammocindole B (**22**)		EC_{50} 6.20 μM*		
	P. vermis	Psammocindole -C (**23**)		EC_{50} >10 μM*		
	Derivative	psammocindole D (**24**)		EC_{50} >10 μM*	Type 2 diabetes	[6]
	Derivative	isopsammocindole A(**25**)	Alkaloids	EC_{50} >10 μM*		
	Derivative	isopsammocindole B(**26**)		EC_{50} >10 μM*		
	Derivative	isopsammocindole C(**27**)		EC_{50} >10 μM*		
	Derivative	isopsammocindole D(**28**)		EC_{50} >10 μM*		
New Guinea, 9-18 m	*P. aff. bulbosa*	Psymberin (**29**)	Polyketide	IC_{50} <25 nM*	colorectal cancer (CRC)	[43]

		**29**		LC_{50}>2.5 x 10^{-5} M*	Leukemia cell lines	
		**29**		LC_{50}<2.5 10^{-9} M*	breast cancer cell lines MDA-MB-435	
		**29**		LC_{50} 1.9 x10^{-5} M*	breast cancer cell line NCI/ADR-RES	
		**29**		LC_{50} 1.36 x10^{-5} M*	Breast cancer cell line T-47D	
		**29**		LC_{50}>2.5 x 10^{-5} M*	Breast cancer cell line MCF7 and HS578T	
New Guinea, x m	*Psammocinia* sp.	**29**		LC_{50}>2.5 × 10^{-5} M*	melanoma cell line (LOX IMVI, UACC-257, SK-MEL-2)	[44]
		**29**		LC_{50} <2.5 × 10^{-9} M*	melanoma cell line (MALME-3M and SK-MEL-5)	
		**29**		LC_{50} 1.41 × 10^{-5} M*	melanoma cell line (SK-MEL-28)	
		**29**		LC_{50} >2.5 x 10^{-5} M*	colon cancer (HT29, SW-620)	
		**29**		LC_{50}<2.5 × 10^{-9} M*	colon cancer (HCT-116)	
		**29**		LC_{50} 3.76 × 10^{-7} M*	colon cancer (HCC-2998)	
Indonesia, 10 m	*Psammocinia* sp.	Cyclocinamide A (**30**)		6% viability**	HCT-116 cancer cells	[46]
New Guinea, 9-18 m	*Psammocinia* sp.	**30**		No activity**	adenocarcinoma cell line	[45]
Indonesia, 10 m	*Psammocinia* sp.	Cyclopsammocinamide A (**31**)		50 µg/mL**	Not active against HCT-116 cells	
		Cyclopsammocinamide B (**32**)		50 µg/mL**	Not active against HCT-116 cells	[46]
New Guinea, 9-18 m	*P. aff. bulbosa*	(-)-preswinholide A (**33**)		Not assayed	-	
		(-)-psymbamide A (**34**)	Polyketide	Not assayed	-	[49]
		DDA (**35**)		Not assayed	Starting material	
		prenyl bromide (**36**)		Not assayed	Starting material	
		intermediate (**37**)		Not assayed	Starting material	
		intermediate (**38**)		Not assayed	Starting material	[49]
Indonesia, x m	*Psammocinia* sp.	Sulawesin A (**39**)	Terpene	IC_{50} 2.7 to 4.6 µM*	ubiquitin-specific protease 7 (USP7)	[51]
		Sulawesin B (**40**)		IC_{50} 2.7 to 4.6 µM*	USP7	
		Sulawesin C (**41**)		IC_{50} 2.7 to 4.6 µM*	USP7	
		ircinin-1 (**42**) and ircinin-2 (**43**)		IC_{50} 2.7 to 4.6 µM*	USP7	
Indonesia, x m	*Spongia* sp.	7*E*,12*E*,20Z,18*S*)-variabilin (**44**)		IC_{50} 1.5 µM*	Inhibitors of protein tyrosine phosphatase 1B	[52]
	Spongia sp.	**44**		IC_{50} 0.8 µM*	protein tyrosine phosphatases T-cell PTP	
	Spongia sp.	**44**		IC_{50} 50 µM*	Non-cytotoxic to Huh-7 and EJ-1	
Indonesia, x m	*Ircinia* sp.	**44**		72% inhibition**	Inhibitors of protein tyrosine phosphatase 1B	[54]
Australia, x m	*Psammocinia* sp.	**44**		Not assayed	-	[55]
South Korea, 20 m	*Ircinia* sp.	**44**		ED_{50} 18.6 to 24.1 µM*	A549, SK-OV-3, SK-MEL-2, XF498, and HCT15	[14]
New Zealand,x m	*Sarcotragus* sp.	**44**		Not assayed	-	[69]
New Zealand, x m	*Ircinia variabilis*	**44**		Not assayed	-	[69]
Indonesia, x m	*Spongia* sp.	(12*E*,20*Z*,18*S*)-8-hydroxyvariabilin (**45**)		IC_{50} 7.1 µM*	Inhibitors of protein tyrosine phosphatase 1B	[52]

	Ircinia sp.	**45**	IC_{50} 3.7 μM*	protein tyrosine phosphatases T-cell PTP	[52]
	Ircinia sp.	**45**	IC_{50} 50 μM*	Non-cytotoxic to Huh-7 and EJ-1	[52]
	Ircinia sp.	**45**	72% inhibition**	Inhibitors of protein tyrosine phosphatase 1B	[54]
	Spongia sp.	furospongin-1 (**46**)	IC_{50} 9.9 μM*	Inhibitors of protein tyrosine phosphatase 1B	[52]
	Spongia sp.	**46**	IC_{50} 9.6 μM*	protein tyrosine phosphatases T-cell PTP	
Indonesia, x m	*Spongia* sp.	**46**	IC_{50} >50 μM*	Non-cytotoxic to Huh-7 and EJ-1	[52]
Australia, 15 m	*Psammocinia* sp.	Ircinialactam H (**47**)	Not assayed	-	[55]
		Ircinialactam I (**48**)	Not assayed	-	
		Ircinialactone A (**49**)	Not assayed	-	
		(7*Z*,12*Z*,20*Z*,18*S*)-variabilin (**50**)	Not assayed	-	
		(7*Z*,12*E*,20*Z*,18*S*)- variabilin (**51**)	Not assayed	-	
		(7*Z*,12*E*,20*Z*,18*S*)-variabilin (**52**)	Not assayed	-	
		Irciniafuran A (**53**)	Not assayed	-	
		Ircinialactam A (**54**)	Not assayed	-	
		Ircinialactam B (**55**)	Not assayed	-	
		Ircinialactam C (**56**)	Not assayed	-	
		Ircinialactam G (**57**).	Not assayed	-	[55]
South Korea, 20 m	*Psammocinia* sp.	Psammocinin A1 (**58**)	ED_{50} 7.5 to >30 μM*	A549, SK-OV-3, SK-MEL-2, XF498, and HCT15	[14]
		Psammocinin A2 (**59**)	ED_{50} 4.8 to 19.3 μM*		
		Psammocinin B (**60**)	ED_{50} 19.2 μM*		
		Palinurin (**61**)	ED_{50} 4.4 to 10.2 μM*		
		Isopalinurin (**62**)	ED_{50} >30 μM*		
		(8*E*,13*Z*,18*R*,20*Z*)-strobilinin (**63**)	IC_{50} 5.8 to 11 μM*		
		(7*E*,13*Z*,18*R*,20*Z*)-felixinin (**64**)	IC_{50} 5.8 to 11 μM*		
		(8*Z*,13*Z*,18*R*,20*Z*)-strobilinin (**65**)	IC_{50} 14.1 to >30 μM*		
		7*Z*,13*Z*,18*R*, 20*Z*)-felixinin (**66**)	IC_{50} 14.1 to >30 μM*		
South Korea, x m	*Psammocinia* sp.	12-deacetoxy-23-hydroxyscalaradial (**67**)	IC_{50} 31.1 to >50 μM*	A498, ACHN, MIA-paca, PANC-1, and CV-1	[5]
		12-dehydroxy-23-hydroxyhyrtiolide (**68**)	IC_{50} 40.7 to >50 μM*		
		12-O-acetyl-16-deacetoxy-23-acetoxyscalarafuran (**69**)	IC_{50} 31.1 to >50 μM*		
South Korea, x m	*Psammocinia* sp.	12-deacetoxy-23-hydroxyheteronemin (**70**)	IC_{50} 0.4 to 0.8 μM*	A498, ACHN, MIA-paca, PANC-1, and CV-1	[5]
South Korea, 15-20 m	*Smenospongia* sp.	70	LC_{50} 0.02 μg/mL*	human leukemia cell line K562	[61]
South Korea, 25-30 m	*Smenospongia* sp.	**70**	LC_{50} 0.13 μg/mL*	human leukemia cell line K562	[62]
		**70**	MIC >0.78 μg/mL*	*Bacillus subtilis* (ATCC 6633)	
		**70**	MIC >100 μg/mL*	*S. aureus* (ATCC 65389)	
		**70**	MIC >100 μg/mL*	*Micrococcus leuteus* (IFC 12708)	

		**70**	MIC >100 μg/mL*	*Proteus Vulgaris* (ATCC 3851)	
		**70**	MIC >100 μg/mL*	*Salmonella typhimurium* (ATCC 14028)	
		**70**	IC_{50} >100 μg/mL*	*In vitro* isocitrate lyase	[62]
South Korea, x m	*Psammocinia* sp.	12-deacetoxy-23-acetoxy-19-O-acetylscalarin (**71**)	IC_{50} 1.2 to 2.1 μM*	A498, ACHN, MIA-paca, PANC-1, and CV-1	[5]
South Korea, 15-20 m	*Smenospongia* sp.	**71**	LC_{50} 4.9 μg/mL*	human leukemia cell line K562	[61]
South Korea, 25-30 m	*Smenospongia* sp.	**71**	LC_{50} 4.9 μg/mL*	human leukemia cell line K562	[62]
		**71**	MIC 3.12 μg/mL*	*Bacillus subtilis* (ATCC 6633)	
		**71**	MIC >100 μg/mL*	*Salmonella typhimurium* (ATCC 14028)	
		**71**	MIC >100 μg/mL*	*S. aureus* (ATCC 65389)	
		**71**	MIC >100 μg/mL*	*E.coli*	
		**71**	MIC >100 μg/mL*	*Micrococcus leuteus* (IFC 12708)	
		**71**	MIC >100 μg/mL*	*Proteus Vulgaris* (ATCC 3851)	
South Korea, 25-30 m	*Smenospongia* sp.	**71**	IC_{50} 4.9 μg/mL*	*In vitro* isocitrate lyase	[62]
South Korea, x m	*Psammocinia* sp.	12-deacetoxy-23-O-acetoxyheteronemin (**72**)	IC_{50} 1.6 to 5.8 μM*	A498, ACHN, MIA-paca, PANC-1, and CV-1	[5]
South Korea, x m	*Psammocinia* sp.	12-deacetoxyscalaradial (**73**)	IC_{50} 38.8 to 48 μM*	A498, ACHN, MIA-paca, PANC-1, and CV-1	[5]
South Korea, 25-30 m	*Smenospongia* sp.	(-)-ircinianin (**74**)	80±10% at 100 μM**	selective α1 GlyR potentiator	[63]
		**74**	IC_{50} 25.4 μM*	*Plasmodium falciparum*	[53]
		**74**	IC_{50} 16.6 μM*	*Leishmania donovani*	
		**74**	No inhibition	Severe Acute Respiratory Syndrome Coronavirus 2	
Australian, 20 m	*Ircinia wistarii*	**74**	No inhibition	human cytomegalovirus	
		**74**	MIC > 32 μg/mL*	ESKAPE panel and a mycobacterial strain	
		**74**	No inhibition	*Litomosoide sigmodontis* (Anthelmintic Assay)	
		**74**	IC_{50} 19.5 μg/mL	human tumour cell line against L6 (non-cytotoxic)	
		**74**	IC_{50} > 64 μg/mL*	HeLa cells (non-cytotoxic)	[53]
Australian, x m	*Psammocinia* sp.	**74**	80 ± 10% μM**	selective α1 GlyR potentiator	[63]
Australian, x m	*Psammocinia* sp.	(-)-ircinianin sulfate (**75**)	IC_{50} 38.4 ± 2.8 μM*	selective α1 GlyR potentiator	[63]
		**75**	IC_{50} 3.2 ±2.1 μM*	selective α3 GlyR potentiator	[63]
		(-)-ircinianin lactam A (**76**)	260 ± 15% at 100 μM**	showed no substantial effect	[63]
Australian, 20 m	*Ircinia wistarii*	**76**	Not assayed	-	[53]
Australian, x m	*Psammocinia* sp.	(-) Ircinianin lactam A sulfate (**77**)	70 ± 7%, at 100 μM**	selective α1 GlyR potentiator	[63]
Australian, 20 m	*Ircinia wistarii*	**77**	Not assayed	-	[53]
	Ircinia wistarii	(-)-oxoircinianin (**78**)	MIC 100 μM	showed no substantial effect	[53]
Australian, 20 m	*Ircinia wistarii*	**78**	Not assayed	-	[53]

	Psammocinia sp.	(-)-oxoircinianin lactam A (**79**)		110 ± 8% at 100 μM**	selective potentiator of α1 GlyRs	[53]
Australian, 20 m	*Ircinia wistarii*	**79**		Not assayed	-	[53]
Australian, x m	*Psammocinia* sp.	(-)-Ircinianin lactone A (**80**)		MIC 100 μM*	showed no substantial effect	[63]
Australian, 20 m	*Ircinia wistarii*	**80**		Not assayed	-	[53]
Australian, x m	*Psammocinia* sp.	(-)-ircinianin acetate (**81**)		MIC 100 μM*	showed no substantial effect	[63]
Australian, 20 m	*Ircinia wistarii*	**81**		Not assayed	-	[53]
Australian, 20 m	*Ircinia wistarii*	ircinianin lactone B (**82**)		Not assayed	-	[53]
Australian, 20 m	*Ircinia wistarii*	ircinianin lactone C (**83**)		Not assayed	-	[53]
	Derivative of **74**	wistarin (**84**)		Not assayed	-	[53]
		insuetolide A (**85**)		MIC 140 μM*	*Neurospora crassa* (fungus)	[65]
		**85**		No inhibition	MOLT-4 human leukemia cell line	
		insuetolide B (**86**)		Not assayed	-	
		insuetolide C (**87**)		51% at 50 μg/mL**	MOLT-4 human leukemia cell line	
Israel, 20 m	*Psammocinia* sp.	4′-hydroxy-2′-butenoyl)-strobilactone A(**88**)		55% at 50 μg/mL**	MOLT-4 human leukemia cell line	
		2α, 9α, 11-trihydroxy-6-oxodrim-7-ene (**89**)		Not assayed	-	
		strobilactone A (**90**)		MIC 242 μM*	*Neurospora crassa* (fungus)	
		**90**		No inhibition	MOLT-4 human leukemia cell line	
		Dihydroxy-2′,4′-octadienoyl)-strobilactone A (**91**)		MIC 162 μM*	*Neurospora crassa* (fungus)	[65]
		**91**		72% at 50 μg/mL**	MOLT-4 human leukemia cell line	
Iran, 10-12 m		GC-MS fractions	PE	MIC 10-20 mg/mL**<br>MBC 20-80 mg/mL**	ESKAPE panel	[67]
Iran, 10-12 m	*Psammocinia* sp.	GC-MS fractions	Carboxylic	90.32% inhibition*	*K. pneumoniae* biofilms	
			Acid	90.86% inhibition*	*P. aeruginosa* biofilms	

Note: x m stands for the unidentified depth of the sample collection in the reports. PE: Phenolic Ester, * full response dose curve, ** single dose screen.
